# Systematic Review and Meta-Analysis of Milk Fat Globule Membrane Supplementation for Mental Well-Being

**DOI:** 10.3390/nu18020342

**Published:** 2026-01-21

**Authors:** Charlotte Mawson, Andrew M. Carroll, Stefanie Evas, Sarah J. Spies, Maher Fuad

**Affiliations:** 1 Fonterra Research and Development Centre, Private Bag 11029, Dairy Farm Road, Palmerston North 4472, New Zealand; charlotte.mawson@fonterra.com (C.M.); andrew.carroll@fonterra.com (A.M.C.); stefanie.evas@adelaide.edu.au (S.E.); johannie.spies@fonterra.com (S.J.S.); 2 Human Health, Health & Biosecurity, Commonwealth Scientific and Industrial Research Organisation (CSIRO), Adelaide 5000, Australia; 3 School of Psychology, Faculty of Health and Medical Sciences, University of Adelaide, Adelaide 5005, Australia

**Keywords:** milk fat globule membrane, MFGM, stress, anxiety, depression, meta-analysis, randomised controlled trial, mood, neuroinflammation, gut–brain axis

## Abstract

**Background/Objectives**: The milk fat globule membrane (MFGM) is a complex structure of polar lipids, gangliosides, and glycoproteins that has demonstrated anti-inflammatory, neuroprotective, and gut-modulatory effects in preclinical and human studies, but its effects on adult psychological outcomes have not been systematically synthesised. **Methods:** We conducted a systematic literature search across multiple databases using combined relevant keywords and Medical Subject Headings terms, with manual reference checks to ensure comprehensiveness. Of the 35 articles initially identified, 3 randomised controlled trials met the inclusion criteria: adult participants (≥20 years); bovine MFGM supplementation; a placebo or control group; and outcomes measuring stress, anxiety, or depression. A random-effects meta-analysis was performed, calculating standardised mean differences for stress, anxiety, and depression outcomes. **Results:** MFGM supplementation produced small but statistically significant reductions in stress and anxiety. Effects on depression were non-significant, though directionally favourable. Risk-of-bias assessments were conducted using Cochrane criteria and indicated low concerns across trials. Publication bias was not indicated, but interpretation was limited by the small number of studies. **Conclusions:** Whilst the evidence for depression is inconclusive, bovine MFGM supplementation may confer modest benefits for stress and anxiety in adults and could be part of a nutritional strategy to support overall mental well-being.

## 1. Introduction

Mental health challenges such as stress and anxiety are increasingly recognised as major global health concerns, affecting individuals across all age groups and socioeconomic backgrounds [1]. Subclinical symptoms of depression, anxiety, and stress can also disrupt daily functioning and overall well-being, with estimates suggesting a substantial proportion of individuals are experiencing subthreshold levels of mental health difficulties [2]. Although stress is a normal physiological response, persistent or elevated levels of stress can impair mental health over time [3]. Mental health conditions arise from a complex interplay of behavioural, environmental, and biological factors, including poor sleep, chronic stress exposure, nutritional deficiencies, and inflammation. While pharmacological treatments such as anxiolytics and antidepressants are commonly used for certain conditions, they often come with side effects such as sedation, sexual dysfunction, and gastrointestinal disturbances [4]. Consequently, there is growing interest in nutritional interventions that may offer safer, complementary approaches to managing stress, anxiety, and depression.

Lifestyle modifications, including regular physical activity, mindfulness practices and dietary changes, are widely recommended for mental well-being [5]. Mental well-being refers to a state in which individuals can manage life’s stresses, realise their abilities, learn and work productively, and participate meaningfully in their communities [6]. Among dietary strategies for mental well-being, bioactive components in functional foods have gained attention for their potential to modulate neurobiological pathways [7]. The milk fat globule membrane (MFGM), a complex structure composed of polar lipids (glycerophospholipids and sphingolipids), gangliosides, and glycoproteins surrounding fat droplets in milk, has emerged as a promising candidate due to its demonstrated anti-inflammatory, gut health, and neuromodulating properties [8,9,10,11]. Importantly, the MFGM is not supplemented as a purified and isolated fraction; rather, interventions typically use ingredients enriched in MFGM, such as buttermilk or cream-derived fractions. These ingredients are often characterised and quantified by their phospholipid content, as phospholipids represent a measurable and well-established proxy for the MFGM-enriched fraction used in nutritional interventions.

Preclinical and clinical studies link MFGM components such as phosphatidylserine, sphingomyelin, and milk fat globule epidermal growth factor 8 (MFG-E8) to attenuated neuroinflammation, improved myelination, and modulation of microglial phenotype [12,13,14]. Together, these effects on inflammation, myelination, and microglial activity may translate into improvements in brain function and health, thereby supporting overall mental well-being and resilience to stress. In addition, MFGM appears to impact the gut–brain axis by shaping microbial composition [15] and associated metabolite profiles [16], which can alter peripheral immune signalling and neuroactive pathways. Furthermore, MFGM supplementation has shown the potential to enhance neuronal complexity and reduce neuroinflammation in animal models [11,17], further supporting its role in brain health and mental well-being.

Despite these promising findings, no meta-analysis has yet systematically evaluated the effects of bovine-derived MFGM on depression, stress, and anxiety outcomes. This meta-analysis aims to synthesise existing evidence from randomised controlled trials to assess the efficacy of MFGM supplementation in supporting mental well-being. By consolidating current research, we seek to clarify the therapeutic potential of MFGM and identify directions for future investigation.

## 2. Materials and Methods

### 2.1. Searching and Selection Processes

A comprehensive literature search in accordance with the Preferred Reporting Items for Systematic Reviews and Meta-Analyses (PRISMA) 2020 statement and checklist [18] (see Supplementary Material) was conducted to identify all relevant studies published up to September 2025. One author (S.J.S.) systematically searched major electronic databases, including PubMed, Scopus, Web of Science, the Cochrane Library, Google Scholar, ACS Publications, Academic Search Index, BMJ Journals, and BNP Media, among others. Only studies published in English were considered. The search strategy combined relevant keywords and MeSH terms such as ‘milk fat globule membrane,’ ‘MFGM, ‘stress’, ‘anxiety’, ‘depression’, ‘mental health’, ‘psychological well-being’, ‘adults’, and ‘randomised controlled trial’, as well as appropriate Boolean operators. To further strengthen the search, the reference lists of all included articles and related reviews were manually checked to identify any additional studies that met the criteria.

The PICO parameters were defined to ensure consistent study selection. The population of interest was adults aged 20 years and older, with no restrictions on sex, ethnicity, or baseline health status. The intervention was bovine-derived MFGM sourced from dairy ingredients such as cream or buttermilk. Eligible studies were required to include an appropriate comparator, as well as either a placebo or a no-supplement control group, and trials without a clearly defined control or those that included a matched phospholipid comparator were not considered. To meet the outcome criteria, studies needed to report at least one measure related to stress, anxiety, or depression. Those lacking any of these psychological outcomes were excluded.

### 2.2. Inclusion and Exclusion Criteria

The inclusion criteria were as follows: (i) designed as randomised controlled trials, either using a parallel-group or crossover format; (ii) participants were aged 20 years or older; (iii) the intervention was a bovine-derived MFGM ingredient; and (iv) the outcomes included at least one psychological outcome related to stress, anxiety, or depression. Studies were excluded if they met any of the following criteria: (i) involvement of special populations such as pregnant women; (ii) lacking a clearly defined control group; (iii) including additional supplements outside the scope of this review; (iv) acute postprandial studies; and (v) consisting of non-human research, review articles, conference abstracts, or publications for which outcome data were unavailable or could not be converted for analysis.

### 2.3. Data Extraction and Quality Appraisal

The titles and abstracts of all identified studies were independently screened by two authors (A.M.C. and S.E.). Full-text articles were obtained for any studies that appeared to meet the inclusion criteria. Any differences in judgement were resolved through discussion or, if required, by consulting a third reviewer. The data was then independently extracted using a standardised template by the same authors. Extracted information included study characteristics (such as author, year of publication, sample size, and study duration), participant demographics, details of the MFGM intervention and corresponding control conditions, and all relevant mental well-being outcomes. Any disagreements in extraction were reconciled by discussion. Where necessary, corresponding authors were contacted to obtain missing information. The risk of bias was evaluated for each trial, using the Cochrane criteria, across seven domains: (i) randomisation, (ii) allocation concealment, (iii) blinding of participants and personnel, (iv) blinding of outcome assessors, (v) selective outcome reporting, (vi) incomplete outcome data, and (vii) other potential sources of bias. Each domain was classified as low risk of bias, some concerns/unclear risk of bias, high risk of bias, or no information. An overall judgement of low risk was assigned only when all assessed domains were at low risk, and conversely, a study was considered high risk if one or more domains were rated as high risk. Two investigators evaluated the potential risks of bias, and any disagreements between reviewers were discussed until consensus was reached.

### 2.4. Measures of Stress, Anxiety, and Depression

The tests covered in this analysis are the Perceived Stress Scale (PSS) and the Depression Anxiety Stress Scale-21 (DASS-21). The perceived stress scale (PSS) is a widely used measure for assessing experienced levels of stress in research settings. This 10-item self-report questionnaire evaluates how often individuals have perceived situations in their lives as uncontrollable, unpredictable, or overwhelming during the preceding month, as well as their perceived ability to manage these stressors. Scores on the PSS range from 0 to 40, with higher scores reflecting greater perceived stress [19,20]. The Depression Anxiety Stress Scale-21 (DASS-21) is a 21-item self-report questionnaire with three domains covering stress, anxiety, or depression, with scores ranging from 0 to 34. Higher scores indicate higher symptomology in the various domains [21]. DASS-S, DASS-A, and DASS-D refer to the stress, anxiety, and depression subdomains, respectively.

### 2.5. Statistical Analysis

The meta-analysis was completed using R Studio (version 4.5.1) to estimate the pooled effect size of the difference between stress, anxiety, and depression final outcomes for MFGM intervention and control [22,23]. The random-effects model was chosen to account for the expected heterogeneity across studies, arising from differences in populations, measurement methods, and intervention differences. The pooled effect size was calculated as a standardised mean difference (SMD), with 95% confidence intervals (CIs), to allow for the pooling of the measures on different scales. The SMD was computed using Hedge’s g to take into account some of the studies’ small sample sizes [24,25]. Effect size of the standardised mean difference was interpreted as follows: 0.2 = small, 0.5 = moderate, and 0.8 = large effect (as per Cochrane guidelines). Heterogeneity was calculated using the DerSimonian and Laird method [26], with Cochran’s Q test and the I2 statistic used to evaluate the proportion of variability in effect sizes beyond chance. Higher values of I2 indicate greater levels of inconsistency, with thresholds of 25%, 50%, and 75% tentatively associated with low, moderate, and high heterogeneity, respectively [27]. Prediction intervals [28] were also included with the pooled result to address the potential underestimation of heterogeneity that can occur with the DerSimonian and Laird method. Publication bias was analysed using funnel plot analysis and Egger’s test [29]. Where studies had multiple intervention arms (high- and low-dose MFGM), the control group for these intervention arms was halved to overcome the unit-of-analysis error that can occur if the same participants are included twice in the analysis.

## 3. Results

### 3.1. Results of the Search and Study Characteristics

**Figure 1:**
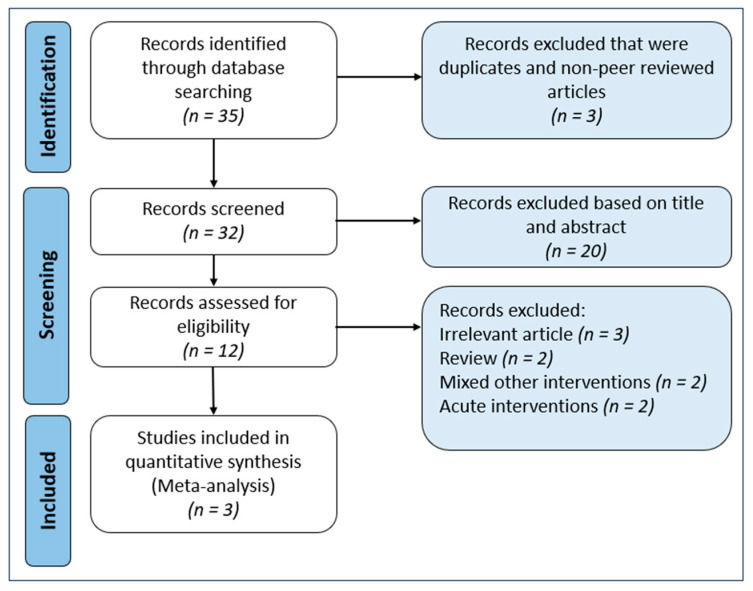
Study selection process based on PRISMA guidelines.

The search strategy yielded 35 articles from various databases. After removing duplicates and non-peer-reviewed studies, 32 studies remained. Screening based on titles and abstracts led to the exclusion of 20 studies, which included animal experiments, non-randomised controlled trials (non-RCTs), reviews, and studies using non-bovine-sourced MFGM or milk as the sole source of MFGM, leaving 12 articles. These 12 articles underwent a full-text review, resulting in the exclusion of 9 articles due to irrelevance (n = 3), being a review article (n = 2), mixed interventions (n = 2), and acute interventions (n = 2). A flowchart of the selection process is presented in Figure 1.

The details of the included studies [30,31,32] are summarised in Table 1. A total of 438 adults participated in studies where MFGM served as the primary dietary intervention. The participants were a mixture of men and women. The studies were published between 2019 and 2025. All three studies were parallel controlled trials. Additionally, two studies included two active arms with high and low doses. The intervention durations ranged from 6 to 16 weeks. Consequently, the doses tested varied widely. All studies reported the phospholipid doses administered to participants, which ranged from 300 mg/day to 4.8 g/day. The choice of control also varied, with the studies using milk protein concentrate with added butteroil, maltodextrin, or rice starch. All studies reported stress measures using either the perceived stress scale (PSS) and/or DASS-21; two studies reported anxiety measures using DASS-21. All studies were conducted in adults aged between 20 years and 75 years. Zajac et al. [32] did not publish the results of the DASS-21, so the authors were contacted directly to obtain these results.

### 3.2. Risk of Bias Assessment

**Figure 2:**
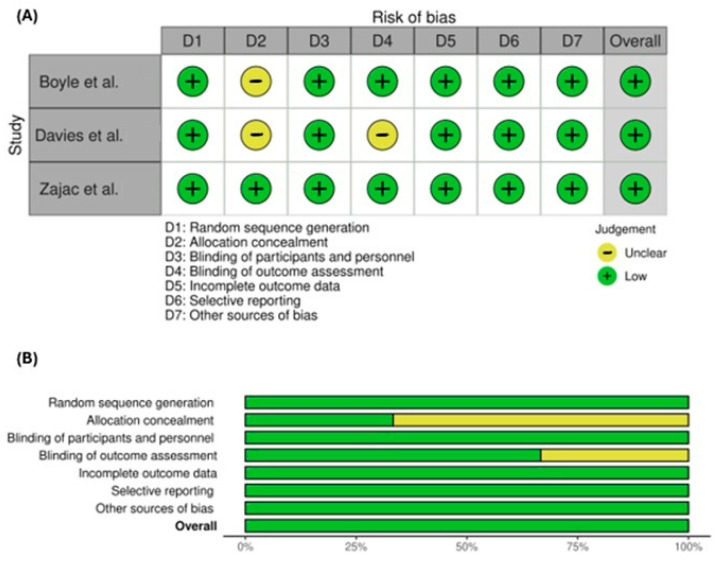
Assessment of bias risk in included studies. Panel (**A**) shows a bias risk summary, with bias risk classified as low (+) or unclear (−) (there were no ratings for high bias risk or no information) [30,31,32]; panel (**B**) shows a bias risk graph (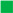, low; 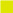, unclear; there were no ratings for high or critical bias risk or for no information) with reviewing authors’ judgements about the bias risk of each item shown as percentages across all included studies. Images were generated using the Robvis tool [33].

Overall, all three trials were rated as low risk. In terms of individual domains, all three trials were classified as having a low risk of bias in random sequence generation. Regarding allocation concealment, one trial was rated as low risk, while two trials lacked sufficient information to determine the risk of bias. In terms of blinding of outcome assessment, one trial was rated as unclear on risk of bias, while the other two trials were rated low risk. All trials were evaluated as having a low risk of bias in the domains of blinding of participants and personnel, incomplete data, selective reporting, and other sources of bias (Figure 2).

### 3.3. Stress

**Figure 3:**
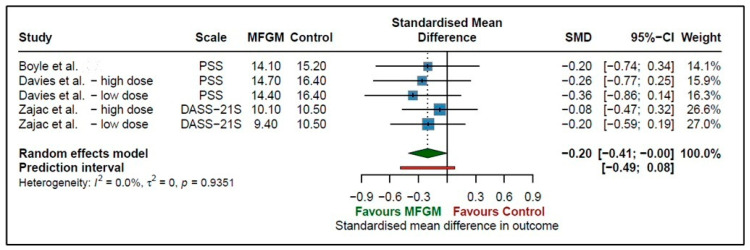
Effect of MFGM supplementation on stress measures in adults [30,31,32].

Three studies investigated the effect of supplementing MFGM on stress outcomes in adults. Two studies [31,32] contributed two dose arms each (high- and low-dose); therefore, five comparisons were included. The pooled results indicate that MFGM may significantly decrease stress, with a small effect size (SMDs = −0.20; 95% CI −0.41 to −0.00, *p* = 0.0495, *I*^2^ = 0%) (Figure 3). The prediction interval (−0.49 to 0.08) crosses the line of no effect, indicating that the effect may vary in future studies, including the possibility of finding no effect on stress outcomes. There was no heterogeneity found (*I*^2^ = 0%, *p* = 0.9351) between studies.

### 3.4. Anxiety

**Figure 4:**
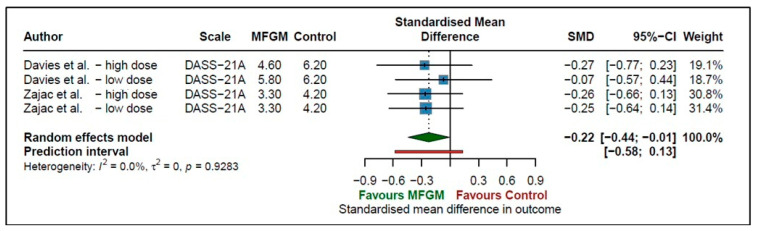
Effect of MFGM supplementation on anxiety measures in adults [31,32].

Two studies [31,32] investigated the effect of supplementing MFGM, both with a high dose or a low dose of MFGM, on anxiety in adults. The pooled standardised mean difference was −0.22 (95% CI −0.44 to −0.01, *p* = 0.0442, *I*^2^ = 0%), indicating a small effect size and, therefore, providing evidence that MFGM may significantly decrease anxiety in adults (Figure 4). The prediction interval (−0.58 to 0.13) indicates that the observed effect could differ in future research, and some studies may report no effect, as the interval crosses zero. There was no heterogeneity found (*I*^2^ = 0%, *p* = 0.9283) between studies.

### 3.5. Depression

**Figure 5:**
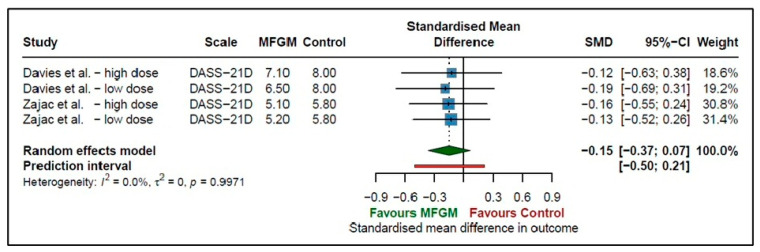
Effect of MFGM supplementation on depression measures in adults [31,32].

Two studies [31,32] investigated the effect of supplementing MFGM, both with a high dose or a low dose of MFGM, on depression in adults. The pooled standardised mean difference was −0.15 (95% CI −0.37 to 0.07, *p* = 0.1839 *I*^2^ = 0%), which indicates the null hypothesis cannot be rejected, and therefore, MFGM was not found to have an effect on depression outcomes in adults (Figure 5). Although the results trend toward improvement in depression, the prediction interval (−0.50 to 0.21) spans zero, suggesting that future studies may yield a range of outcomes, including no effect. There was no heterogeneity found (*I*^2^ = 0%, *p* = 0.9971) between the studies.

### 3.6. Heterogeneity Measure

Although no heterogeneity was found between the studies for stress, anxiety, or depression, with the limited number of studies included, this value should be interpreted with caution [34].

### 3.7. Publication Bias

**Figure 6:**
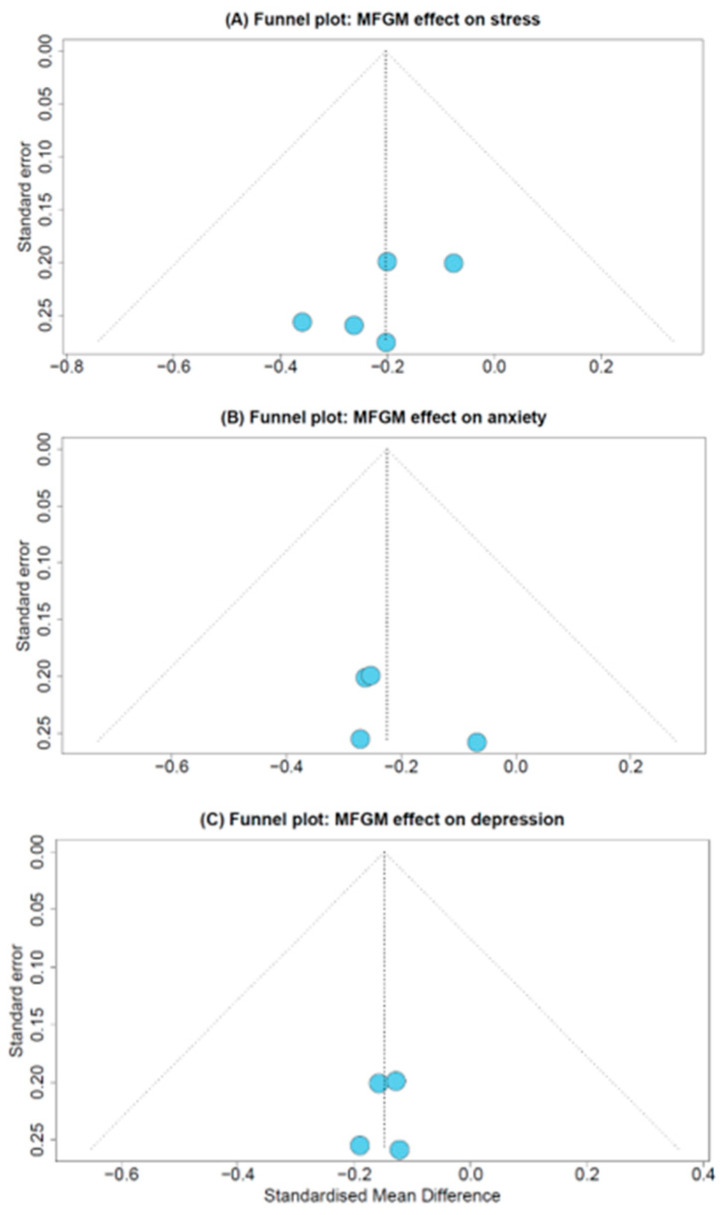
Funnel plots for (**A**) MFGM and stress, (**B**) MFGM and anxiety, and (**C**) MFGM and depression.

Funnel plots were utilised to evaluate publication bias (Figure 6). In meta-analyses with less than ten studies, the interpretation of funnel plots must be treated with caution, as it can be difficult to determine asymmetry. Additionally, the power attributed to Egger’s test is reduced. Therefore, these results do not provide conclusive evidence to elucidate the risk of publication bias. However, the Egger regression test for funnel plot asymmetry was not statistically significant for stress (*p* = 0.235), anxiety (*p* = 0.416), or depression (*p* = 0.751) measures, and no obvious asymmetry can be seen in the three funnel plots.

## 4. Discussion

This meta-analysis evaluated the effect of MFGM supplementation on adult mental health outcomes across a range of populations. Synthesising data from three eligible studies, we found that MFGM was associated with statistically significant improvements in anxiety and stress measures, whereas effects on depression were not statistically significant but directionally favourable. Importantly, the observed effects of MFGM (SMD ≈ 0.20–0.22) are comparable with some pharmacotherapies for mental well-being [35], highlighting the practical relevance of these findings and indicating its promise as a potential dietary intervention for mental well-being. However, it is important to note that the populations included in this analysis are not clinically diagnosed, and therefore, this comparison needs to be interpreted in this context. Overall, these findings suggest that MFGM supplementation may offer modest benefits for improving stress and anxiety in adults and provide a nutritional strategy to support mental well-being.

The MFGM is a complex dietary fraction composed of polar lipids, gangliosides, and glycoproteins that has demonstrated anti-inflammatory, neuroprotective, and gut-modulatory effects in preclinical studies and human research [36]. These biological activities provide a plausible mechanism for the effects of MFGM on mental well-being, including reductions in stress and anxiety. The convergence of immune, neural, and gut mechanisms makes the MFGM a particularly interesting candidate for interventions aimed at stress-related disorders, which are increasingly understood as multisystem conditions [37]. 

There are several hypotheses that link MFGM-containing ingredients with mental well-being benefits. A central pathophysiological pathway linking MFGM to mental health is the hypothalamic–pituitary–adrenal (HPA) axis. The HPA axis is the central neuroendocrine system that drives cortisol release in a circadian and feedback-regulated manner [38], and chronic inflammation can lead to persistent activation of the axis [39]. Chronic inflammation and persistent HPA activation are common features of stress, anxiety, and depressive disorders, with inflammatory cytokines such as IL-6 and TNF-α frequently elevated in those with anxiety and stress disorders [40]. MFGM has demonstrated anti-inflammatory activity in preclinical studies and some human studies [8,41,42], and by attenuating systemic inflammation, it could reduce persistent inflammatory drive to the HPA axis, helping to normalise cortisol dynamics and stress responses. Restored HPA regulation would be expected to enhance emotional regulation and stress resilience.

Peripheral inflammation can also influence the brain via other routes. Inflammatory signals can prime microglia toward a proinflammatory phenotype, leading to cytokine release within the brain and subsequent neuroinflammation [43]. In preclinical models, MFGM components, including the glycoprotein MFG-E8, promote microglial phagocytosis and shift microglia toward an anti-inflammatory M2 phenotype, with downstream effects on reducing neuroinflammatory signalling [14]. Supplementation with MFGM containing ingredients has also been shown to decrease microglial activation and neuroinflammation caused by a high-fat diet [44]. Regarding translation to humans, these actions could reduce neuroinflammatory signalling and, through this, support better mood regulation and stress responses. Additionally, neuroinflammation can also impair myelin integrity, and emerging evidence links reduced myelination with depression disorders [45]. Myelin is the protective sheath around nerve fibres that enables rapid and efficient nerve impulse transmission. Damage or abnormalities in this sheath can disrupt communication between brain regions, which is now recognised as a key factor in mood- and stress-related disorders. MFGM has been found to support brain myelination in infants in motor-related regions of the brain; however, evidence in adults is limited [46]. By supporting myelination, MFGM may enhance neuronal communication and enable efficient and synchronised brain function, key elements integral to mood regulation and cognitive processing under stress [47].

The gut–brain axis offers a further complementary route. MFGM has been associated with favourable shifts in the microbiome, enhanced gut barrier function and altered microbial metabolite profiles in preclinical studies and some human studies [15,16]. By reducing intestinal permeability and limiting translocation of proinflammatory microbial products, such as lipopolysaccharide, MFGM may lower peripheral inflammation further, as well as HPA activation [9,48]. Additionally, modulation of microbial communities and their metabolites can influence central neurotransmitter systems and vagal signalling; MFGM has been found to enhance serotonin production by lactic acid bacteria, suggesting one plausible microbe-mediated path to improved mood and stress resilience [16]. MFGM has also been found to support cognitive function in an Alzheimer’s disease mouse model by modulating gut bacteria, which, in turn, increased butyrate production, activating the FFAR2 signalling axis [49]. However, these proposed mechanisms remain hypotheses that require validation in future studies. It is also important to note, as previously mentioned, that MFGM is not supplemented as an isolated component in clinical trials. Rather, it is provided through ingredients enriched in MFGM, which contain a broader range of bioactive compounds in addition to MFGM itself. These additional components may also contribute to the observed effects and therefore should not be discounted.

A key strength of this analysis is the explicit inclusion of randomised evidence and the use of standardised mean differences to enable cross-study comparability across diverse outcome measures. The use of a random-effects framework for the anxiety/stress outcome appropriately acknowledges the likely heterogeneity in populations, intervention protocols, and outcome instruments. The clear reporting of prediction intervals provides a sense of the range of effects that might be observed in new studies, underscoring the uncertainty inherent to the current evidence base.

While this meta-analysis provides valuable insights, several limitations should be acknowledged. Firstly, the small number of eligible studies (n = 3) restricts the generalisability of the findings. Secondly, the included trials tested a wide range of daily MFGM phospholipid doses (approximately 300 mg to 4.0 g/day), varied in duration (6–16 weeks), and used different control arms. The heterogeneity in exposure complicates inference about an optimal dose or minimum effective dose. The control groups differed substantially in composition (e.g., milk protein + butteroil, maltodextrin, rice starch), which introduces additional heterogeneity and makes direct physiological comparison more challenging. This variability in control composition limits the ability to attribute observed effects solely to MFGM-enriched ingredients. Additionally, all studies relied on self-reported measures of psychological outcomes, which are susceptible to measurement error and reporting bias. Despite these limitations, the findings nonetheless indicate that MFGM supplementation may hold promise for improving aspects of mental well-being. Further studies are required to expand the evidence base and allow for more precise effect estimates and reliability of heterogeneity and publication bias. Future studies should aim to design studies with similar control groups to enable comparability of the studies. Exploration of dose–response relationships, trial duration, and potential moderators (age, sex, baseline mood, or stress levels and diet) would help identify subgroups most likely to benefit from supplementation. Mechanistic studies investigating inflammatory markers, neurotrophic factors, and gut–brain axis mediators could also help to clarify the biological pathways underlying any mood-related effects of MFGM.

## 5. Conclusions

In conclusion, this meta-analysis indicates that MFGM supplementation was associated with statistically significant improvements in anxiety and stress measures. While effects on depression remain inconclusive, the consistent benefits observed warrant broader consideration of MFGM as part of integrative dietary strategies for mental health. The limited number of studies and the small effect sizes underscore the need for further well-designed trials to confirm the consistency, magnitude, and clinical relevance of these findings.

## Figures and Tables

**Table 1:** Description of studies included in this meta-analysis.

Author	Country	Age Range, Mean or Median	Participants (Number in Total and in Each Arm)	Study Design	Intervention	Intervention Dosage (Phospholipids)	Control Group	Intervention Duration	Outcomes
Boyle et al. [30]	England	Mean of 22 ± 0.8 years in MFGM, mean of 20.8 ± 0.3 years in placebo	53 perfectionist healthy males: 26 in MFGM group, 27 in control group	Parallel, two arms	Milk protein concentrate and MFGM in a water-based drink per day	2.7 g phospholipids	Milk protein concentrate with added butteroil (0 mg phospholipids) in a water-based drink per day	6 weeks	Stress (PSS)
Davies et al. [31]	New Zealand	Mean of 37.2 ± 9.9 years in MFGM (high-dose), mean of 38.8 ± 11 years in MFGM (low-dose), mean of 39.1 ± 8.6 years in placebo	122 healthy men and women with moderate levels of stress: 30 in MFGM high-dose, 32 in MFGM low-dose, 60 in the control group	Parallel, three arms	Low-dose buttermilk-derived MFGM and high-dose buttermilk-derived MFGM mixed with water, milk or as a smoothie per day	Low-dose: 300 mg phospholipids High-dose: 600 mg phospholipids	Maltodextrin mixed with water, milk or smoothie per day	12 weeks	Stress (DASS-21S, PSS), Anxiety (DASS-21A), Depression (DASS-21D)
Zajac et al. [32]	Australia	≥55 and ≤76 years across MFGM and placebo groups	236 healthy men and women with self-reported memory complaints 78 in MFGM high-dose, 79 in MFGM low-dose and 79 in the control group	Parallel, three arms	Skim milk powder fortified with low-dose buttermilk-derived MFGM and high-dose buttermilk-derived MFGM mixed with 200 mL of water per day	Low-dose: 1.7 g phospholipids High-dose: 4.0 g phospholipids	50 g rice starch mixed with 200 mL of water per day	16 weeks	Stress, anxiety and depression (DASS-21S, DASS-21A, DASS-21D)

## Data Availability

No new data were created or analysed in this study. Data sharing is not applicable to this article.

## Supplementary Materials

The following supporting information can be downloaded at: https://www.mdpi.com/article/10.3390/nu18020342/s1. PRISMA 2020 Checklist.
